# Extensive Pneumomediastinum, Pneumopericardium, and Subcutaneous Emphysema Complicating Anti-melanoma Differentiation-Associated Gene 5 (Anti-MDA5)-Positive Dermatomyositis-Associated Fibrosing Interstitial Lung Disease: A CT-Based Case Report

**DOI:** 10.7759/cureus.110217

**Published:** 2026-06-03

**Authors:** El Mahdi Choukri, Anas Tahtah, Ali Meqor, Zakaria Boulahcen, Salma Erguiague, Maroua Haddadi, Adib Remmal, Bouchra Dahmani, Siham Alaoui Rachidi

**Affiliations:** 1 Diagnostic and Interventional Radiology, Mohammed VI University Hospital, Tangier, MAR; 2 Radiology, Faculty of Medicine and Pharmacy, Abdelmalek Essaâdi University, Tangier, MAR

**Keywords:** anti-mda5 dermatomyositis, computed tomography, fibrosing ild, interstitial lung disease, pneumomediastinum, pneumopericardium, subcutaneous emphysema, traction bronchiectasis

## Abstract

Dermatomyositis-associated interstitial lung disease may be severe, particularly in patients with anti-melanoma differentiation-associated gene 5 (anti-MDA5) antibody-positive disease, and can be complicated by air-leak phenomena. We report the case of a 60-year-old woman with newly diagnosed anti-MDA5-positive dermatomyositis-associated interstitial lung disease who was admitted for worsening dyspnea and extensive subcutaneous emphysema. Clinical examination revealed diffuse subcutaneous crepitus involving the thorax, neck, anterior chest wall, including the breasts, and back, bilateral pulmonary crackles, Gottron papules, erythematous skin lesions, and proximal lower-limb weakness.

Non-contrast thin-section chest CT demonstrated diffuse chronic fibrosing interstitial lung disease with peripheral and basal predominance, manifested by septal and non-septal reticulations, intralobular reticulation, honeycomb-like fibrotic change, traction bronchiectasis, and persistent basal ground-glass opacities on expiratory images, as well as bilateral pulmonary micronodules. CT also showed extensive pneumomediastinum, pneumopericardium, right pleural or extrapleural air extension, and diffuse subcutaneous emphysema extending into the cervical, thoracic, axillary, and deep facial soft tissues. Limited upper abdominal sections showed hepatic, splenic, and pancreatic uncinate process calcifications, considered incidental in the context of the thoracic examination. The patient was treated with high-dose corticosteroids and immunosuppressants, including mycophenolate mofetil and cyclophosphamide, with prophylactic anticoagulation. This report highlights the value of CT in mapping air-leak complications and characterizing the underlying fibrosing interstitial lung disease in anti-MDA5-positive dermatomyositis.

## Introduction

Dermatomyositis is an idiopathic inflammatory myopathy that may involve the lungs, with interstitial lung disease being one of its most clinically important extramuscular manifestations [1]. The radiological spectrum of dermatomyositis-associated interstitial lung disease is broad and may include ground-glass opacities, reticulation, traction bronchiectasis, and fibrotic remodeling, which are most often assessed using high-resolution CT [2-4]. Recognition of these findings is essential, as pulmonary involvement can significantly influence prognosis and guide therapeutic decisions.

Pneumomediastinum is an uncommon but potentially serious complication in patients with dermatomyositis-associated interstitial lung disease. It may occur in the setting of fragile alveolar structures and fibrosing parenchymal disease, leading to air dissection along bronchovascular sheaths into the mediastinum through the Macklin effect and, in extensive cases, into the cervical and subcutaneous soft tissues [5-7]. Anti-melanoma differentiation-associated gene 5 (anti-MDA5) antibody-positive dermatomyositis is particularly associated with severe interstitial lung disease and has been linked to the development of pneumomediastinum in myositis-associated interstitial lung disease [8,9]. This association is clinically relevant because pneumomediastinum in anti-MDA5-positive dermatomyositis-associated interstitial lung disease may indicate more severe pulmonary involvement and has been associated with poorer outcomes in published series [8,9].

We report the case of a 60-year-old woman with newly diagnosed anti-MDA5-positive dermatomyositis-associated interstitial lung disease who presented with worsening dyspnea and extensive air-leak complications. Chest CT demonstrated chronic fibrosing interstitial lung disease complicated by extensive pneumomediastinum, pneumopericardium, right pleural or extrapleural air extension, and diffuse subcutaneous emphysema. This report highlights the critical role of CT in the comprehensive assessment of acute respiratory deterioration in patients with connective tissue disease-associated interstitial lung disease.

## Case presentation

A 60-year-old woman was admitted to the internal medicine department in January 2025 for worsening dyspnea in the context of newly diagnosed anti-MDA5-positive dermatomyositis-associated interstitial lung disease. Her symptoms had begun approximately five months earlier with progressive asthenia, polyarthralgia, an erythematous skin eruption affecting both hands and the thorax, and bilateral eyelid edema. Before admission, she had received oral prednisone, methotrexate, and folic acid.

On admission, the patient was conscious and clinically stable, with oxygen saturation of 96% on room air, heart rate of 81 beats per minute, and respiratory rate of 19 breaths per minute. Physical examination revealed diffuse subcutaneous emphysema involving the thorax, neck, anterior chest wall, including the breast regions, and back, with palpable crepitus. Bilateral pulmonary crackles were heard up to the mid-lung fields. Cardiac auscultation was unremarkable.

Cutaneous examination showed Gottron papules and erythematous lesions on the elbows and great toes. Neuromuscular examination revealed a slow and difficult gait, proximal pelvic girdle weakness, and lower-limb motor weakness. There were no signs of respiratory muscle failure, choking episodes, swallowing disorder, dysphagia, or chest pain. Pulmonary function testing performed before admission showed a restrictive ventilatory defect, with vital capacity at 64% of predicted and an FEV1/FVC ratio (Tiffeneau index) of 89%. A previous chest CT performed in December 2024 had revealed dissecting subcutaneous emphysema, pneumomediastinum, a small right pneumothorax, bronchiectatic changes, and interstitial lung disease.

Initial laboratory testing showed mild leukopenia, without anemia or thrombocytopenia. Inflammatory markers were not markedly elevated, and renal, hepatic, and coagulation parameters were within or close to the reference ranges. The main laboratory findings are summarized in Table 1.

**Table 1:** Initial laboratory findings on admission

Laboratory parameter	Result	Unit	Reference range
White blood cell count	3.34	×10³/µL	4-10
Neutrophils	1.92	×10³/µL	1.5-7
Lymphocytes	1.14	×10³/µL	1-4
Hemoglobin	12.1	g/dL	12-16
Platelet count	232	×10³/µL	150-450
C-reactive protein	5	mg/L	<6
Erythrocyte sedimentation rate, first hour	28	mm	<30
Serum creatinine	6	mg/L	6-11
Aspartate aminotransferase	29	U/L	<31
Alanine aminotransferase	31	U/L	<34
Prothrombin activity	100	%	70-100
International normalized ratio	1.00	-	Reference value: 1
Activated partial thromboplastin time ratio	1.06	-	<1.2

A repeat non-contrast chest CT scan was performed on January 22, 2025, using helical acquisition with thin-section reconstructions. Intravenous contrast was not administered, as the study aimed to assess the lung parenchyma and air-leak complications, including pneumomediastinum and subcutaneous emphysema, which are well visualized on non-contrast thin-section CT. The scan demonstrated diffuse chronic interstitial lung disease with a predominantly peripheral and basal distribution. The abnormalities included septal and non-septal reticulations, intralobular reticulation, focal honeycomb-like fibrotic changes, and bilateral traction bronchiectasis. Persistent basal ground-glass opacities were also seen on expiratory images. Bilateral pulmonary micronodules with centrilobular and peripheral distribution were additionally noted.

Multiplanar lung-window CT images showed extensive pneumomediastinum, pneumopericardium, and diffuse subcutaneous emphysema associated with fibrosing interstitial lung disease. The parenchymal abnormalities included subpleural reticulation/honeycomb-like fibrotic change, traction bronchiectasis, ground-glass opacities, and band-like atelectatic opacity (Figure 1).

**Figure 1:**
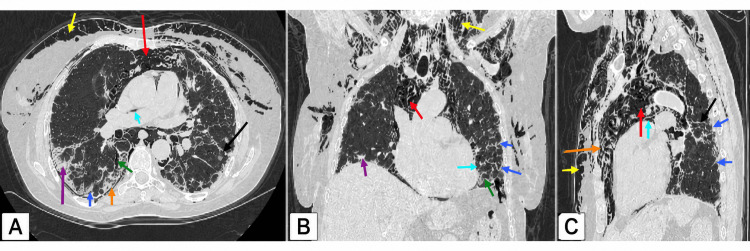
Multiplanar lung-window chest CT showing dermatomyositis-associated fibrosing interstitial lung disease complicated by extensive pneumomediastinum, pneumopericardium, and diffuse subcutaneous emphysema Axial (A), coronal (B), and sagittal (C) lung-window CT images demonstrate extensive pneumomediastinum (red arrows) associated with diffuse subcutaneous emphysema involving the thoracic and cervical soft tissues (yellow arrows). Pneumopericardium is also visible, outlining the pericardial sac (cyan arrows). Background fibrosing interstitial lung disease is characterized by peripheral and basal subpleural reticulation/honeycomb-like fibrotic change (blue arrows), traction bronchiectasis (green arrows), and ground-glass opacities (purple arrows). Band-like atelectatic opacity is indicated by black arrows. Associated pleural or extrapleural air extension is also seen (orange arrows) CT: computed tomography

Axial non-contrast mediastinal-window CT images demonstrated periaortic extension of pneumomediastinum along the descending thoracic aorta, associated right pleural or extrapleural air extension, and multiple upper abdominal calcifications involving the liver, spleen, and pancreatic uncinate process (Figure 2).

**Figure 2:**
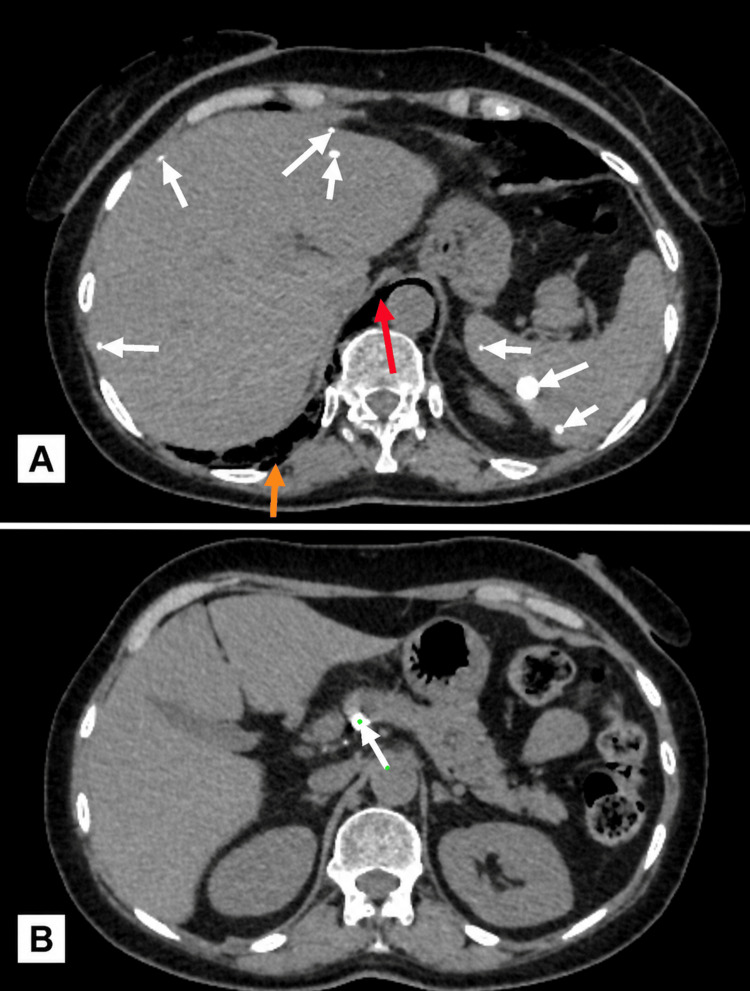
Axial non-contrast CT images showing periaortic pneumomediastinum extension, right pleural or extrapleural air extension, and upper abdominal calcifications Axial non-contrast mediastinal-window CT image (A) demonstrates air tracking along the descending thoracic aorta, consistent with periaortic extension of pneumomediastinum (red arrow), with associated right pleural or extrapleural air extension posteriorly (orange arrow). Multiple hepatic and splenic calcifications are also visible on the included upper abdominal section (white arrows). A lower axial image (B) shows pancreatic uncinate process calcification (white arrow) CT: computed tomography

Inspiratory and expiratory lung-window CT images demonstrated persistent basal ground-glass opacities associated with chronic fibrosing interstitial abnormalities, including subpleural reticulation/honeycomb-like change, traction bronchiectasis, and band-like atelectatic opacity. Associated air-leak findings were also visible where present (Figure 3).

**Figure 3:**
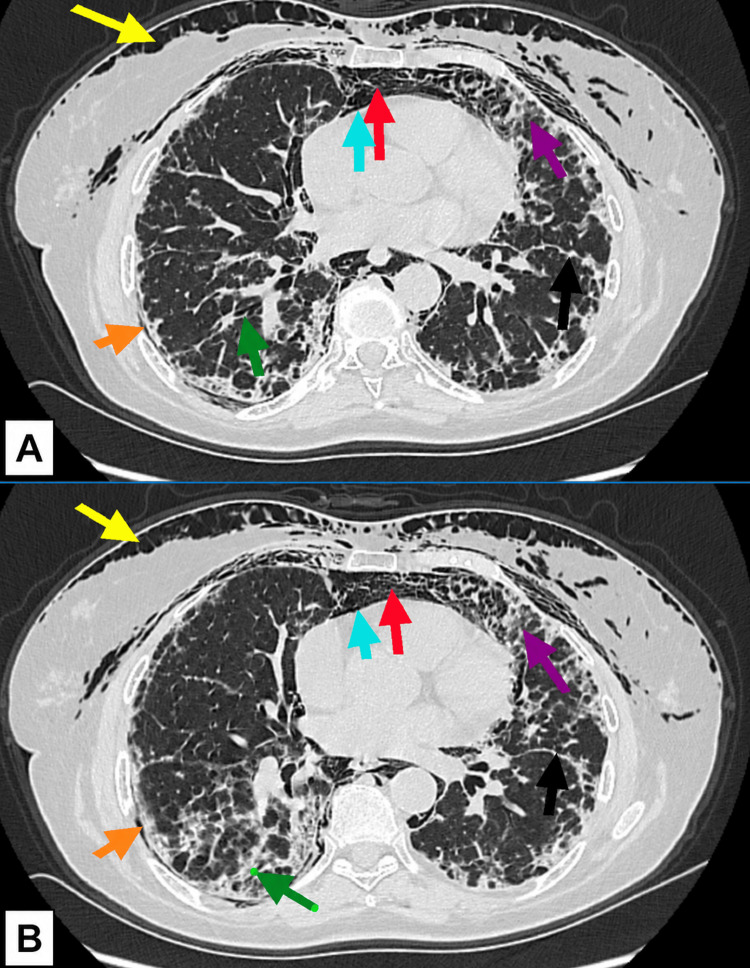
Inspiratory and expiratory lung-window CT showing persistent basal ground-glass opacities in dermatomyositis-associated fibrosing interstitial lung disease Inspiratory (A) and expiratory (B) lung-window CT images demonstrate persistent basal ground-glass opacities (purple arrows), associated with chronic fibrosing interstitial abnormalities including subpleural reticulation/honeycomb-like fibrotic change (blue arrows), traction bronchiectasis (green arrows), and band-like atelectatic opacity (black arrows). Associated air-leak findings are also visible where present, including pneumomediastinum (red arrows), subcutaneous emphysema (yellow arrows), pneumopericardium (cyan arrows), and pleural or extrapleural air extension (orange arrows) CT: computed tomography

The air dissection extended into the cervical, axillary, and thoracic soft tissues, reaching the deep facial spaces at the upper limit of the acquired images. There was no significant mediastinal lymphadenopathy, although a few subcentimeter cervical lymph nodes were noted. There was no pleural or pericardial effusion. No acute osseous abnormalities were identified on the acquired images. During hospitalization, the patient was treated with intravenous methylprednisolone boluses followed by oral corticosteroid therapy. Immunosuppressive treatment included mycophenolate mofetil and cyclophosphamide, with the first cyclophosphamide course administered on January 23, 2025. Prophylactic anticoagulation was also administered. The patient was discharged after a 15-day hospitalization with a planned follow-up for the second cyclophosphamide course.

Overall, the CT findings were consistent with chronic fibrosing interstitial lung disease associated with anti-MDA5-positive dermatomyositis, complicated by extensive pneumomediastinum, pneumopericardium, right pleural or extrapleural air extension, and diffuse subcutaneous emphysema. The hepatic, splenic, and pancreatic uncinate process calcifications were considered incidental findings on the limited upper abdominal sections.

At the time of manuscript preparation, no follow-up CT documenting radiological resolution was available. The available clinical course was limited to the index hospitalization, after which the patient was discharged with planned follow-up for the second cyclophosphamide course.

## Discussion

Dermatomyositis is an idiopathic inflammatory myopathy with potentially severe extramuscular involvement, particularly interstitial lung disease. Pulmonary disease may dominate the clinical course and can range from inflammatory ground-glass-predominant abnormalities to chronic fibrosing interstitial lung disease with reticulation, traction bronchiectasis, and honeycombing [1-4]. In the present case, CT demonstrated a chronic fibrosing pattern with peripheral and basal predominance, including septal and non-septal reticulations, intralobular reticulation, focal honeycomb-like fibrotic changes, traction bronchiectasis, and persistent basal ground-glass opacities. These findings support a fibrosing form of dermatomyositis-associated interstitial lung disease rather than an isolated acute air-leak event.

Pneumomediastinum is an uncommon but clinically important complication of dermatomyositis-associated interstitial lung disease. The proposed mechanism is commonly explained by alveolar rupture with air tracking along the bronchovascular sheaths toward the mediastinum, known as the Macklin effect [5]. In patients with fibrosing interstitial lung disease, architectural distortion, traction injury, cough, increased intra-alveolar pressure, and fragile subpleural or peribronchovascular alveoli may contribute to air leakage. Once air reaches the mediastinum, it can extend into the cervical fascial planes, chest wall, axillary regions, and, in extensive cases, toward the deep facial spaces. This mechanism explains the extensive pneumomediastinum and diffuse subcutaneous emphysema observed in our patient.

The association between dermatomyositis and pneumomediastinum has been reported for several decades, but it remains rare [6,7]. More recent studies have emphasized its prognostic relevance in myositis-associated interstitial lung disease, particularly in patients with anti-MDA5-positive dermatomyositis [8,9]. In the present case, anti-MDA5 antibody positivity was documented, supporting the diagnosis of anti-MDA5-positive dermatomyositis-associated interstitial lung disease. This serological context is clinically important because anti-MDA5-positive disease is frequently associated with severe pulmonary involvement and may be complicated by extensive air-leak manifestations.

CT is central to diagnosis because it simultaneously evaluates both components of the disease: the underlying interstitial lung disease pattern and the extent of extra-alveolar air. In this case, thin-section non-contrast CT allowed identification of fibrosing interstitial abnormalities, including traction bronchiectasis and focal honeycomb-like fibrotic change, while lung-window and mediastinal-window images demonstrated extensive air dissection involving the mediastinum, periaortic region, pericardial region, cervical soft tissues, thoracic wall, and axillary soft tissues. CT also helps assess associated complications such as pneumothorax, pleural or extrapleural air extension, pleural effusion, pericardial effusion, mediastinal compression, and signs of tension physiology.

From a clinical perspective, pneumomediastinum in dermatomyositis-associated interstitial lung disease should not be considered a benign incidental finding, especially when it is extensive or associated with worsening dyspnea. Published data suggest that pneumomediastinum may be associated with more severe disease and poorer outcomes in myositis-associated interstitial lung disease [8,9]. In selected severe cases, tension pneumomediastinum may lead to cardiorespiratory compromise and require urgent intervention [10]. Therefore, radiological reporting should clearly describe the volume and distribution of pneumomediastinum, its cervical and subcutaneous extension, associated pleural or extrapleural air, pneumopericardium when present, the presence or absence of pneumothorax, and any signs of mediastinal compression.

Limited upper abdominal sections showed hepatic, splenic, and pancreatic uncinate process calcifications, with the splenic calcifications described according to a CT pattern-recognition approach [11]. These findings were considered incidental in the context of the thoracic CT examination and were not interpreted as directly related to dermatomyositis-associated interstitial lung disease or pneumomediastinum. Because the acquisition was limited to the thoracic CT field, no comprehensive abdominal characterization was possible.

The management of pneumomediastinum depends on clinical severity, respiratory status, and the underlying interstitial lung disease activity. Conservative measures may be sufficient in stable patients, including oxygen therapy, analgesia, rest, cough control, and close monitoring. However, patients with anti-MDA5-positive dermatomyositis-associated interstitial lung disease often require multidisciplinary management involving internal medicine, pulmonology, rheumatology, radiology, and intensive care teams when respiratory deterioration occurs. In the present case, treatment was directed mainly toward the underlying inflammatory myopathy-associated interstitial lung disease and included high-dose corticosteroid therapy, mycophenolate mofetil, and cyclophosphamide. The air-leak complications required careful clinical and radiological monitoring.

This case report highlights three important teaching points. First, worsening dyspnea or diffuse subcutaneous crepitus in anti-MDA5-positive dermatomyositis-associated interstitial lung disease should prompt careful evaluation for air-leak complications, including pneumomediastinum, pneumopericardium, pleural or extrapleural air extension, pneumothorax, and subcutaneous emphysema. Second, CT is the key imaging modality because it accurately maps both fibrosing parenchymal disease and the extent of extra-alveolar air. Third, when pneumomediastinum is identified in this context, the report should be structured and clinically actionable, with explicit mention of pneumothorax, pleural or extrapleural air extension, pericardial involvement, cervical and subcutaneous extension, and signs of tension physiology.

## Conclusions

This report highlights extensive pneumomediastinum, pneumopericardium, pleural or extrapleural air extension, and diffuse subcutaneous emphysema as clinically important air-leak complications of anti-MDA5-positive dermatomyositis-associated fibrosing interstitial lung disease. In patients with connective tissue disease-associated interstitial lung disease presenting with worsening dyspnea or diffuse subcutaneous crepitus, CT is essential for identifying air-leak complications, defining their extent, and assessing the underlying fibrosing parenchymal abnormalities. A structured radiological evaluation should include the volume and distribution of pneumomediastinum, cervical and subcutaneous extension, pleural or extrapleural air, pneumothorax, pericardial involvement, and any signs of mediastinal compression. Incidental upper abdominal calcifications may also be detected on limited thoracic CT sections and should be reported cautiously, taking into account the available field of view and clinical context. Early recognition of these findings can guide multidisciplinary management and close clinical monitoring.
